# Panton-Valentine leukocidin in community and hospital-acquired *Staphylococcus aureus* strains

**DOI:** 10.1080/13102818.2014.976457

**Published:** 2014-11-07

**Authors:** Tuncer Özekinci, Tuba Dal, Keramettin Yanık, Nida Özcan, Şükran Can, Alicem Tekin, Halil İbrahim Yıldırım, İdris Kandemir

**Affiliations:** ^a^ Department of Medical Microbiology, Faculty of Medicine, Dicle University, Diyarbakır, Turkey; ^b^ Department of Medical Microbiology, Faculty of Medicine, Yıldırım Beyazıt University, Ankara, Turkey; ^c^ Department of Medical Microbiology, Faculty of Medicine, Ondokuz Mayıs University, Samsun, Turkey; ^d^ Department of Medical Microbiology, Ergani State Hospital, Diyarbakır, Turkey; ^e^ Department of Genetics, Faculty of Veterinary Medicine, Dicle University, Diyarbakır, Turkey

**Keywords:** *Staphylococcus aureus*, Panton-Valentine leukocidin, MRSA, MSSA

## Abstract

*Staphylococcus aureus* causes serious hospital-acquired (HA) and community-acquired (CA) infections. Skin and soft-tissue infections especially are sometimes caused by strains harbouring Panton-Valentine leukocidin (PVL). PVL belongs to a family of bi-component leukocidal toxins produced by staphylococci. It is a pore-forming toxin encoded by *lukF-PV* and *lukS-PV*. A total of 70 *S. aureus* strains: 38 (54%) methicillin-resistant (MRSA) and 32 (46%) methicillin-susceptible (MSSA), were isolated from patients admitted to Dicle University Hospital (Turkey). Identification of *S. aureus* and antibiotics-susceptibility testing were performed with PHOENIX 100. PVL genes and *mecA* genes were detected by polymerase chain reaction. Of the 70 studied strains, 36 ones (51%) were community acquired and 34 ones (49%) were hospital acquired . A total of 38 (54%) strains were positive for *mecA* (*mecA* ^+^), of which 32 ones (84%) were HA. Of the *mecA* ^−^ strains, 30 (94%) were CA. Of the 70 studied strains, 12 (17%) strains were PVL^+^: 8 (22%) of the 36 CA strains and 4 (12%) of the 34 HA strains. Of the 12 PVL^+^ strains, 4 strains were *mecA* ^+^. The PVL positivity rate was 25% in MSSA, whereas 10.5% in MRSA. Of the overall PVL^+^ strains, seven strains were obtained from wounds; four ones from skin abscess; and one from blood culture. Taken together, the obtained results showed a substantial level of PVL genes in the studied region. Although PVL is known as a common virulence factor of CA MRSA, HA MRSA isolates in our study showed a considerable rate of PVL positivity.

## Introduction

*Staphylococcus aureus* can cause infections ranging from local skin and soft-tissue infections to life-threatening diseases, such as bacteremia and necrotizing pneumonia. *S. aureus* is one of the most prevalent pathogens that causes serious hospital-acquired (HA) and community-acquired (CA) infections.[1]

Methicillin-resistant *S. aureus* (MRSA) was first described in 1961 in England [2] and has become endemic and epidemic in hospitals worldwide.[3] MRSA isolation rates increased in the United States, Asia and some European countries and Turkey.[1,3,4] In *S. Aureus*, methicillin resistance caused by the production of a low affinity penicillin-binding protein (PBP2a) is encoded by the *mecA* gene, located on a large mobile genetic element – the staphylococcal chromosomal cassette *mec* (*SCCmec*).[5,6] Whereas CA MRSA strains usually harbour *SCCmec* type IV and V, HA MRSA strains are mainly associated with *SCCmec*-I, II and III throughout the world.[7,8]

*S. aureus* infections, especially skin and soft-tissue infections, are sometimes caused by strains harbouring Panton-Valentine leukocidin (PVL), which belongs to a family of bi-component leukocidal toxins produced by staphylococci. PVL is a pore-forming toxin encoded by *lukF-PV* and *lukS-PV* [9] and is known as a virulence factor sometimes associated with tissue necrosis.[7] PVL can trigger neutrophil lysis or apoptosis and tissue necrosis by the release of cytotoxic lysosomal granule contents from lysed neutrophils.[10,11] Both methicillin-susceptible *S. aureus* (MSSA) and CA-MRSA can express PVL.[9]

We aimed to determine the rates of PVL in CA or HA *S. aureus* strains isolated from different clinical samples in our hospital.

## Subjects and methods

### Patients

Seventy patients admitted to Dicle University Hospital in 2012 took part in our study. They were from 0 to 79 years of age. Thirty-seven (53%) of the patients were female and 33 (47%) were male. Informed consent was obtained from all patients or their parents/legal guardians.

### Strains

A total of 70 *S. aureus* strains: 38 (54%) MRSA and 32 (46%) MSSA were isolated from clinical samples obtained from the patients. The strains were stored at −80 °C until use. Identification and antibiotics-susceptibility testing were performed with PHOENIX 100 (Becton Dickinson, Franklin Lakes, NJ) and methicillin resistance of strains was confirmed by the cefoxitin disk diffusion test (Oxoid, Hampshire, England), based on recommendations of the Clinical Laboratory Standards Institute. For quality control, reference strains ATCC 43300 and ATCC 29213 and a PVL^+^ strain were used in the study.

### DNA extraction

A boiling technique was used for rapid DNA extraction. Briefly, a fresh passage of the strains was performed and whole colonies were suspended into 500 μL of sterile distilled water and then vortexed. The suspension was incubated in a dry heating block at 100 °C for 15 min and centrifugated at 15000 × *g* for 20 min at 4 °C. Then, 200 μL of supernatant [12] was collected and the DNA concentration was measured by a NanoDrop 1000 spectrophotometer (Thermo, USA). The extracted DNA samples were stored at −20 °C until use.

### *MecA* and PVL polymerase chain reaction (PCR)

Primer sequences for the PVL genes [13] were as follows: forward for *luk-PV-1*, 5′-ATC ATT AGG TAA AAT GTC TGG ACA TGA TCC A-3′; reverse for *luk-PV-2*, 5′-GCA TCA ACT GTA TTG GAT AGC AAA AGC-3′; and for *mecA* [14]: forward 5′-AAA ATC GAT GGT AAA GGT TGG C-3′, reverse 5′-AGT TCT GCA GTA CCG GAT TTG C-3′.

Amplification was performed with a Veriti™ 96-Well Thermal Cycler (Applied Biosystems, CA, USA). PCR conditions were as follows: 5 min at 94 °C, followed by 30 amplification cycles, each consisting of 30 s of denaturation at 94 °C, 30 s of annealing at 55 °C and 1 min of extension at 72 °C. Final extension was performed at 72 °C for 10 min. PCR products were separated electrophoretically in a 1.5% agarose gel in 0.5× TBE (Tris–Borate–Ethylene diamine tetra acetic acid) buffer and photographed (BIO-RAD, Italy). Analyses were done by comparison with a 100 bp GeneRuler (Thermo, Lithuania). DNA fragments of 433 and 310 bp were considered as positive for the *lukS/F-PV* and *mecA* gene, respectively, by comparing with the positive controls.

## Results and discussion

PVL is a biocomponent synergohymenotropic cytotoxin associated with furunculosis, severe necrotizing hemorrhagic pneumonia, necrotizing fasciitis and other lesions involving the skin and mucosa.[15] The gene encoding PVL is frequently found in CA-MRSA strains carrying *SCCmec*-IV.[16] Since the PVL gene is known to be associated with virulence,[17] determining the presence of the PVL gene in MRSA strains might be important to early and proper therapy for serious MRSA infections.[18,19]

**Figure 1.**
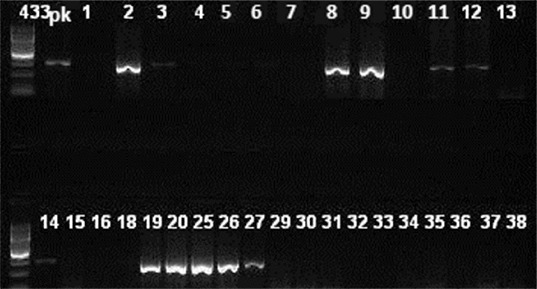
Agarose gel imaging of amplified *lukS*/*F-PV* gene. Lane pk is positive control. DNA fragments of 433 bp (isolates 2, 3, 8, 9, 11, 12, 14, 19, 20, 25, 26 and 27) were considered positive.

**Figure 2.**
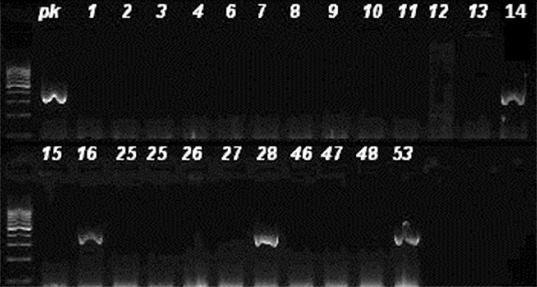
Agarose gel imaging of amplified *mecA* gene. Lane pk is positive control. DNA fragments of 310 bp (isolates 14, 16, 28 and 53) were considered positive.

In our study, the mean age of the patients was 40.96 years and their median age was 43.00 with a standard deviation of ±20.77. Out of the 70 studied strains, 36 strains (51%) were CA and 34 strains (49%) were HA. A total of 30 strains were isolated from wounds, 14 from blood, 12 from sputum, 9 strains were isolated from abscess, 2 from vaginal sample, 2 from urine and 1 from a nasal swap. Of the 70 strains, 38 ones (54%) were positive for *mecA* (*mecA* ^+^), while 32 ones (46%) were negative for *mecA*. Eight (22%) of the CA strains and 30 (88%) of the HA strains were *mecA* ^+^. Of the 38 *mecA* ^+^ strains, 30 (79%) were HA. Of the 32 *mecA* ^−^ strains, 30 (94%) were CA. Twelve (17%) of the 70 studied strains were PVL^+^: 8 (22%) of the 36 CA strains and 4 (12%) of the 34 HA strains (8 CA, 4 HA). Of the 12 PVL^+^ strains (Figure 1), 4 strains were *mecA* ^+^ (Figure 2). Among the methicillin susceptible strains, the PVL positivity rate was 25% (8/32) and among the methicillin resistant ones, 10.5% (4/38). Of the PVL^+^ strains, seven strains were obtained from wounds, four from skin abscess and one from blood culture. The characteristics of the PVL^+^ and *mecA* ^+^ strains are presented in Table 1.

**Table 1.** Characteristics of PVL ^+^ *S. aureus* strains isolated in Dicle University Hospital (Diyarbakır, Turkey).

No.	PVL	*mecA*	Clinical sample	Hospital clinic/polyclinic	HA/CA	Age (years)	Gender
1.	+	−	Abscess	Dermatology P.	CA	29	Female
2.	+	+	Wound	Obstetrics and Gynaecology C.	HA	33	Female
3.	+	−	Wound	Orthopaedic Surgery C.	HA	15	Male
4.	+	−	Wound	Dermatology P.	CA	43	Female
5.	+	−	Wound	Dermatology C.	CA	33	Female
6.	+	−	Wound	Dermatology P.	CA	35	Male
7.	+	−	Wound	Physical Med. and Reh. P.	CA	62	Male
8.	+	−	Abscess	Dermatology P.	CA	45	Male
9.	+	+	Blood	Orthopaedic surgery C.	HA	14	Female
10.	+	+	Abscess	Dermatology C.	HA	60	Female
11.	+	+	Wound	Dermatology P.	CA	38	Female
12.	+	−	Abscess	Otorhinolaryngology P.	CA	19	Male

Note: P.: polyclinic; C.: clinic; CA: community acquired; HA: hospital acquired; and Physical Med. and Reh.: Physical medicine and rehabilitation.

The analysis of our results showed that the rates of PVL genes in the studied region are at a substantial level. Although PVL is known as a common virulence factor of CA MRSA, HA-MRSA isolates in our hospital had a considerable rate of PVL positivity.

Van der Meeren et al. [20] reported 15.1% prevalence of HA-MRSA infection among inpatients as compared to 1% prevalence of CA-MRSA infection among outpatients. In their study, the PVL toxin gene was detected in 81.1% of MSSA and in 11.1% of MRSA. Montagnani et al. [21] reported three cases of severe infections in infants; they were caused by PVL^+^ *S. aureus* and evolved with a positive outcome. Wang et al. [22] observed that the positivity rates of *mecA*, *ermA*, *ermB* and *ermC* in the *S. aureus* isolates were 13/60. Among the 60 isolates, 30 harboured enterotoxin genes, with *sea* being the most frequent toxin gene (33%), followed by *sec* (15%), *sed* (12%) and *seb* (5%). The PVL gene was detected in four strains. Eleven MRSA isolates were of the *SCCmec* type III. Haider et al. [23] reported a case of severe necrotizing haemorrhagic pneumonia in a 12-year-old boy, who needed full ventilatory support and died despite all efforts. In their case presentation, post-mortem examination of lung swabs confirmed the presence of PVL-associated *S. aureus*. Mariem et al. [24] characterized 69 MRSA strains isolated from two Tunisian hospitals and reported that 79% of CA-MRSA strains and 51% of HA-MRSA strains were PVL-positive. According to AlFouzan et al. [25], out of 291 *S. aureus* isolates, 30.6% were MRSA. Genes for PVL were detected in 14.6% and 12.0% of the MRSA and MSSA isolates, respectively. The majority of the PVL-producing MRSA and MSSA were isolated from cases of skin (30.7%) and soft tissue (21.8%) infections. Both MRSA types carried *SCCmec* type III, IV, IVc and V genetic elements.[25] In another meta-analysis including 76 studies from 31 countries, PVL strains were strongly associated with skin and soft-tissue infections, while comparatively rarely with pneumonia, musculoskeletal infections, bacteremias and colonizing strains.[26]

In Turkey, the carriage rate of the PVL gene by MRSA isolates appears to be very low. Karahan et al. [27] reported that out of 304 studied *S. aureus* strains (230 HA and 74 CA), 261 were MRSA and 43 were MSSA. PVL positivity was determined in 12 (11 CA) strains. Eight were MRSA, and four were MSSA. Their results indicated that PVL-positive strains were able to cause infection in nearly every system, without the need for additional risk factors.

Kilic et al. [28] collected 385 clinical MRSA isolates and overall, *SCCmec* types I, II, III, IV, V, nontypeable and PVL occurrence were detected in 11 (2.8%), 3 (0.8%), 316 (82.1%), 20 (5.1%), 20 (5.1%), 15 (3.9%) and 5 (1.3%) isolates, respectively. The PVL gene was detected in 10% of *SCCmec*-IV/V isolates, in contrast to 0.3% in *SCCmec*-I/II/III (χ^2^ = 25.164, *p* < 0.001). Baykam et al. [29] isolated *S. aureus* from anterior nares of 121 patients. MRSA was isolated from 1.2% of these patients and all of the MRSA isolates were positive for the *mecA* and PVL genes. In the study of Tekeli et al. [30], among 100 MRSA bloodstream isolates, the dominant MRSA clone had *SCCmec* type III, *agr* type 1 and revealed sequence type (ST) 239. Alp et al. [31] reported that the rate of MRSA in patients with apparent infections (sepsis, meningitis, lung abscess or septic arthritis) ranged from 12% to 75%, within the seven participating centres. None of the isolates contained the PVL genes. Sesli Çetin et al. [32] obtained nasal and throat swabs from subjects who did not have prior history of any health care exposure. Genotyping of 5 PVL^+^ isolates by pulse field gel electrophoresis revealed that one child and a teacher from the same class were colonized with the clonally related strains, suggesting that close contact with colonized people could be considered a risk factor for being colonized. In the study of Baran et al. [33], 30 strains were phenotypically identified as MRSA and after assessing the risk factors, 28 (93.3%) of them were classified as HA and 2 (6.7%) of them as CA. PVL gene positivity was detected only in CA-MRSA isolates (2/2; 100%). In the report of Akoğlu et al. [34], all the MRSA strains isolated from patients at intensive care units and surgical wards were positive for the *mecA* gene. Of the isolates, 61.8% harboured *SCCmec* type III, 34.5% *SCCmec* variant IIIB and 2.7% *SCCmec* type IV. PVL was positive in 12.7% of the isolates.[34] Demir et al. [35] analysed 92 CA and 150 HA isolates and identified 77 strains as *mecA* positive. PVL was not observed among the MRSA isolates, but 8 (5.3%) HA-MSSA and 14 (15.2%) CA-MSSA, mostly isolated from furuncles (71.4%), were positive for PVL.

Taken together, the results from our study indicated that PVL is an important virulence factor for skin and soft tissue infection caused by *S. aureus*. A limitation of our study was the low number of studied strains. We did not determine the relationship between the PVL positivity and the *SCCmec* types, and also the antibiotic susceptibility rates. We could suggest that large-scale molecular studies associated with *SCCmec* typing of *S. aureus* strains and determining the virulence factors of *S. aureus* strains are necessary for the management of *S. aureus* infections in the studied region.

## Conclusions

In our study, 17% of the analysed *S. aureus* strains were PVL^+^. PVL prevalance was 22% among CA strains and 12% among HA strains. PVL positivity rate was 25% in MSSA, and 10.5% in MRSA isolates. Most of the PVL positive strains were isolated from cutaneous infections, except one blood culture isolate, indicating that PVL could be considered an important virulence factor for skin and soft tissue infection caused by *S. aureus*. The rates of PVL genes in the studied region were shown to be substantial. Although PVL is known as a common virulence factor of CA MRSA, HA-MRSA isolates in our hospital had a considerable rate of PVL positivity. We could suggest that larger scale molecular studies associated with *SCCmec* typing of *S. aureus* strains and determining the virulence factors of *S. aureus* strains are necessary for management of *S. aureus* infections in the studied region.
